# Traditional Cultural Practices Provide More Frequent Feelings of Happiness and Calm for Native American Elders

**DOI:** 10.1093/geroni/igaa057.1087

**Published:** 2020-12-16

**Authors:** Collette Adamsen, Ramona Danielson

**Affiliations:** 1 University of North Dakota, Grand Forks, North Dakota, United States; 2 North Dakota State University, Fargo, North Dakota, United States

## Abstract

Though American Indian, Alaska Native, and Native Hawaiian (AI/AN/NH) elders are an understudied population, available data demonstrates higher rates of depressive symptoms among these elders. In addition, AI/AN/NH elders are a medically underserved population, with geographic isolation a common barrier to accessing emotional/mental health services. However, cultural practices are important sources of resilience for AI/AN/NH elders. Survey data from “Identifying our Needs: A Survey of Elders” Cycle VI (2014-2017), conducted by the National Resource Center on Native American Aging, were analyzed using ordinal logistic regression; N=18,134 adults age 55+ from 164 tribal survey sites. Respondents indicated how often (from none to all of the time) they participate in cultural practices (e.g., traditional food, music, customs). Frequency of participation varied; 27% of elders reported participating in traditional cultural practices a good bit of the time or more, 28% some of the time, 18% a little of the time, and 27% did not ever participate. We explored the relationship between frequency of cultural practices and frequency of feelings of happiness, calm/peacefulness, nervousness, and being downhearted/blue. A significant positive association was found between higher frequency of cultural participation and feelings of happiness and calm/peacefulness; no association was found with nervousness or depressive symptoms. While the frequency of participation by elders in cultural practices is directly related to better self-reported levels of happiness and peacefulness, nearly half (45%) never/almost never participated in these types of practices, which underscores the need to support availability of and elders’ participation in cultural practices.

